# The aerobic mitochondrial ATP synthesis from a comprehensive point of view

**DOI:** 10.1098/rsob.200224

**Published:** 2020-10-21

**Authors:** Alessandro Maria Morelli, Silvia Ravera, Isabella Panfoli

**Affiliations:** 1 Pharmacy Department (DIFAR), Biochemistry Laboratory, University of Genova, Viale Benedetto XV, 3, 16132 Genova, Italy; 2 Experimental Medicine Department (DIMES), University of Genova, Via De Toni, 14, 16132 Genova, Italy

**Keywords:** ATP synthase, mitochondria, endoplasmic reticulum, myelin, oxidative phosphorylation, extra-mitochondrial

## Abstract

Most of the ATP to satisfy the energetic demands of the cell is produced by the F_1_F_o_-ATP synthase (ATP synthase) which can also function outside the mitochondria. Active oxidative phosphorylation (OxPhos) was shown to operate in the photoreceptor outer segment, myelin sheath, exosomes, microvesicles, cell plasma membranes and platelets. The mitochondria would possess the exclusive ability to assemble the OxPhos molecular machinery so to share it with the endoplasmic reticulum (ER) and eventually export the ability to aerobically synthesize ATP in true extra-mitochondrial districts. The ER lipid rafts expressing OxPhos components is indicative of the close contact of the two organelles, bearing different evolutionary origins, to maximize the OxPhos efficiency, exiting in molecular transfer from the mitochondria to the ER. This implies that its malfunctioning could trigger a generalized oxidative stress. This is consistent with the most recent interpretations of the evolutionary symbiotic process whose necessary prerequisite appears to be the presence of the internal membrane system inside the eukaryote precursor, of probable archaeal origin allowing the engulfing of the α-proteobacterial precursor of mitochondria. The process of OxPhos in myelin is here studied in depth. A model is provided contemplating the biface arrangement of the nanomotor ATP synthase in the myelin sheath.

## 1. Introduction

Continual chemical energy supply in the form of ATP is vital for cellular function. Several catabolic pathways are involved in ATP production, even though the enzyme F_1_F_o_-ATP synthase (ATP synthase) remains responsible for most of its generation during the aerobic catabolism of glucose [1]. ATP synthase is coupled to the electron transport chain in the oxidative phosphorylation (OxPhos) pathway [2]. The electrons coming from the reduced coenzymes, namely NADH and FADH_2_, are delivered to molecular oxygen by the four respiratory complexes. The considerable amount of energy gathered from the electron transport chain (ETC) generates a proton current, which is coupled to an ATP synthase nanomachine for ATP production.

The OxPhos machinery was originally identified in the mitochondria; however, in the last years, several authors have reported a functional expression of the ETC and the ATP synthase in other cellular membranes [3–9]. It is important to point out that mitochondria are also fundamental for the extra-mitochondrial OxPhos activity, because, in humans, mitochondrial genome codifies 13 subunits of the respiratory complexes, necessary to guarantee an efficient and complete OxPhos metabolism [10]. We have observed that the extra-mitochondrial OxPhos is performed by the same proteins codified by the mitochondrial DNA [10], which stands in for the OxPhos machinery to be assembled in the mitochondria before its transfer to other membranes to conduct the extra-mitochondrial OxPhos. In the light of these data, it appears that the cellular energy production, pivotal for any cell, can be sustained by other membranous structures besides mitochondria, sometimes bearing a higher ATP synthetic ability than mitochondria, as is the case for the rod outer segments of photoreceptors [11–13]. In the extra-mitochondrial sites, the ATP synthase nanomachines can synthesize aerobic ATP without the restraints imposed by the double membrane system of the mitochondrion. In some cases, the extra-mitochondrial OxPhos was shown to occur on a single membrane, such as the plasma membrane of a cell, suggesting that a closed vesicle or a double membrane system as in mitochondria is not necessary to handle protons (H^+^). Notably, the presence of a close relationship among mitochodria and the endoplasmic reticulum (ER) evidenced by imaging data allows us to suppose that the mitochondrial reticulum and the ER function together in healthy cells, while when this relationship is lost, the aerobic capacity of the cell is diminished [14–19]. A series of analytical data evidence that the ER is the site of the high operability of OxPhos with associated aerobic ATP synthesis. This process is in line with the proposed model for the evolution of mitochondria evolved from bacteria [20–23]. Moreover, many aspects of aerobic ATP synthesis are still unclear.

## 2. A novel perspective on mitochondria evolution

The origin of mitochondria has been widely debated and recent years have witnessed the correction of the original theory as proposed in the 1970s by Margulis & Bermudes [24]. It had been assumed that the mitochondria derive from ancestral α-protobacteria engulfed by archaea cells, then the bacteria would have evolved into mitochondria that express the OxPhos machinery, typical of these. The origin of the eukaryotic host lineage for the mitochondrial endosymbiont, the so-called three-domains–eocyte may as well need revising [25]. Recent phylogenetic analyses found eukaryotic genes within the archaea, and in turn, eukaryotic genomes also comprise archaeal- and bacterial-specific genes [25]. Although links for the prokaryote-to-eukaryote transition are still missing, it is believed that eukaryotes emerged either from a common ancestor with archaea or from these. Possibly, eukaryotes emerged from within the archaeal ‘TACK’ superphylum [20]. The pivotal issue is whether the symbiosis has occurred among bacteria and archaea already endowed with a developed internal membrane system. The archaeal ancestor of eukaryotes might have been more complex than the archaeal lineages identified thus far [20]. The identification of archaeal genes involved in vesicular trafficking indicates that cellular complexity had already emerged before the acquisition of the mitochondrial endosymbiont, as pointed out by Spang *et al*. [20] with the article ‘Complex archaea that bridge the gap between prokaryotes and eukaryotes'. Conceivably, the ancestor of eukaryotes needed dynamic phagocytic capabilities to allow the invagination of the mitochondrial progenitor. Membrane fusion uses the small molecular weight G-protein Rab type already present in archaea [26]. Notably, Rab is present in archaea but not in bacteria. In the proto-eukaryote, Rab would have allowed the incorporation of the OxPhos machinery coming from the engulfed bacteria or have promoted a close collaboration among the pre-existing ER and the newly engulfed protobacteria. This is a crucial point: the engulfed bacteria point to send the OxPhos machinery to the great surface development of the archaea for the more efficient operation of the OxPhos process. However, Rab proteins are expressed in the mature mitochondria [27,28] where they allow considerable increase in the internal surface, i.e. the formation of the crystae. This appears a true symbiosis, as the mitochondria gave access to the OxPhos machinery to the eukaryotic endomembranes and the neo-eukaryote ‘instructed’ the mitochondria to develop the cristae, i.e. to considerably increase the surface of the inner membrane, increasing the efficiency of the OxPhos process. The cristae formation is functional to better functioning of membrane H^+^ capacitor for better functionality of ATP synthase [29]. The H^+^ handling was also analysed and it was found that bacterial phospholipid esters enable better H^+^ lateral currents when in a monolayer over water than the isoprenoid phospholipid ethers of archaea [30]. This is confirmatory of H^+^ currents on the membrane for energy transfer.

**Figure 1.**
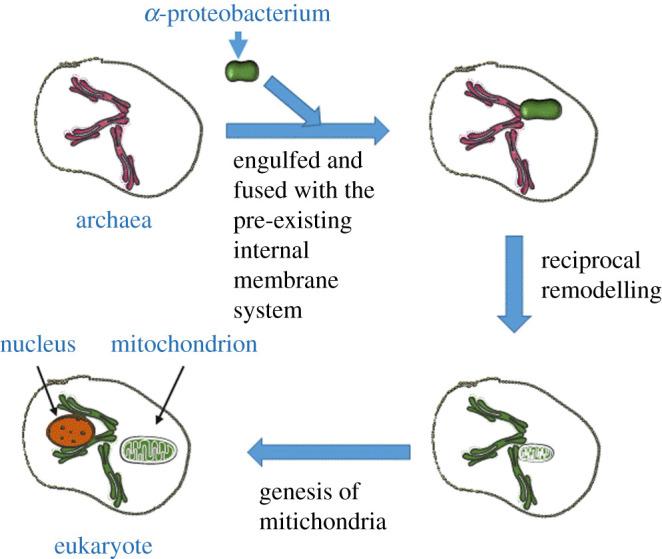
Updated possible mitochondria evolution: the archaea, which displayed a development of internal membranes, engulfed the bacterium expressing the OxPhos machinery, coming in contact with the inner membranes of the archaea. There is a reciprocal remodelling (the inner membranes achieve the OxPhos machinery) and the bacterium acquires from the archaea the molecular devices (small molecular weight-G-protein *in primis*) that determine remodelling and the bacterium forms the cristae, rendering the aerobic ATP synthesis more efficient. The symbiosis with bacteria generates the nucleus in the archaea [28].

It is possible to note that the process of increased internal surface development has also occurred in the opposite direction, i.e. from proto-eukaryote to mitochondria, in fact, the mitochondrion possesses cristae that the bacteria do not have. Figure 1 schematically illustrates these possible processes. Among other many recent contributions, worth mentioning are the studies of Pittis & Gabaldón [21] with the commentary from Ettema [22] where the ‘mito-intermediate’ hypothesis is considered as the most plausible. In short, it is proposed that the archaea cell has developed the internal membrane system before the mitochondrial endosymbiosis. The theory has been further refined [23], hypothesizing that the ancestral bacterium found in the advantageous host cell, the internal membrane development that we now know as the ER, in turn favouring the constant and possible fusion with the mitochondrion. The existence of multiple contact sites among the ER and the mitochondria, called mitochondria-associated membranes (MAMs), has been long known [17]. MAMs are the site of exchange of lipids, calcium (Ca^2+^) and metabolites between these organelles, with an important role in cellular bioenergetics and cell fate [31]. The mitochondria in close proximity to the ER assume a tubular conformation, different form the almost spherical shape they display when the supposed mitochondrial reticulum is disrupted [32,33]. Notably, the classical mitochondrial dyes always stain a network that resembles the ER whose continuity is disrupted when, for example, fusion proteins are defective or the cells have severe aerobic metabolism defects [34].

Interestingly, a study in yeast aiming to determine the origin, either archaeal or bacterial of 25 open reading frame (ORF) groups, inferred the origin of the functional eukaryotic categories. It was found that the organization of the cytosol and the plasma membrane and lipid metabolism were of bacterial origin [27]. By contrast, of archaeal origin were categories related to the nucleus, and ER organization, to the assembly of protein complexes and others. Obviously, mitochondria-related ORF originated from the α-proteobacterial progenitor of mitochondria. Thus, it seems that the ultimate novelty to the eukaryote, as the result of the endosymbiosis with the α-protobacterium is the close relationship among two membranes, i.e. those of the pre-existing ER and that of the mitochondrion, which had not been there before. It was even supposed that the eukaryotic nucleus might originate from a symbiosis in which archaea would become bacterial parasites and, after discarding their original genes, turn into cell nuclei [27]. This seems plausible, given that the nuclear membrane is an extension of the ER.

## 3. Proton movements in the respiring membrane

H^+^ cycling is pivotal to chemical energy conversion. The mode of coupling of the process of the electron transport to the aerobic ATP synthesis has been investigated in depth for more than 60 years, having required a huge investigative effort with hard discussion among scientists. Since the 1960s, it has been accepted that coupling takes place according to the chemiosmotic theory [35], but the foundations of this theory have been questioned and new coupling ways have been proposed as new metabolic, kinetic and proteomic experimental data have accumulated [36–40].

Mitchell introduced the concept of H^+^-motive force (pmf), typically ranging around 250 mV, which would be contributed to by the transmembrane electrical potential difference (Δ*ψ*) plus the pH difference between the two aqueous phases (ΔpH). However, it has been observed that the biological membrane surface is separated from the bulk aqueous phase by ordered water molecules representing an electrostatic barrier, which for H^+^ ranges around 120 meV [41]. Therefore, the value of pmf should be calculated surface-to-surface, rather than bulk-to-bulk pmf. The concept according to which H^+^ translocation fuelling the ATP synthase occurs between aqueous sources and sinks may be reconsidered [42]. In the aqueous bulk, H^+^ exists as hydronium ions. The dielectric/desolvation penalty (greater than 500 meV) for transferring these H^+^ into the hydrophobic membrane phase should be taken into account. There is no evident source of energy for the ATP synthase to carry the H^+^ over the cited desolvation barrier [41]. We have proposed that the transfer of H^+^ occurs along the membrane surface. H^+^ would bind the phospholipid head groups [42]. Along this vision, theoretically, any membrane can be considered as a ‘proton-sponge’ on the basis of the available pH-buffers in the form of phospholipids, which with their phosphate residue can store and dispose of protons at the membrane–cytosol interface [43,44]. The crucial role of the bacterial phospholipid ester to allow the H^+^ current which entails interesting evolutionary considerations was recently highlighted [30]. On the other hand, the existence of H^+^ conductors [45–47] and of H^+^ microcircuits in membranes has been highlighted [48]. H^+^ would reside inside the respiring membrane always bound to a proteolipid-phase or to the respiring complex, never being transferred to water [49]. However, the process of ATP synthesis—that is the ADP + P_i_ the endergonic reaction that provides ATP + H_2_O—does occur in the aqueous phase [42,50,51]. Consistently, the F_1_ moiety of ATP synthase protrudes about 10 nm in the cytosol from the inner mitochondrial membrane (IMM) surface. Such phase separation in turn sets the need for a coupling of the two processes. A nanomechanical coupling would operate inside the respiring membranes. The negative charges of phosphate groups would lie on both sides of the IMM, whereas the positive ones would reside at the non-polar centre of the membrane, where the hydrocarbon tails are compact. The contours for possible membrane H^+^ microcircuits were recently described in detail [36].

## 4. Membrane shielding

**Figure 2.**
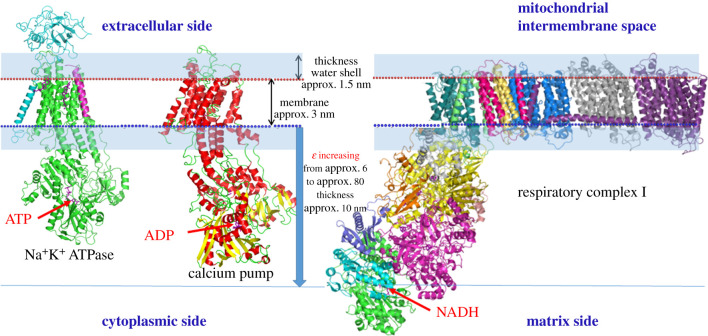
Schematic of macromolecular membrane pumps binding ATP (*a*) and respiratory complex I binding NADH (*b*), embedded in the membrane. The protective layer of water adhering to the membrane and the protrusion for about 10 nm of the zone with low dielectric constant are indicated.

An intriguing reality emerges: a shielding of the membrane limits the interaction of ionic species dissolved in the cellular aqueous medium with the membrane surface, covered by a layer of water molecules which thickness is estimated around 1.5 nm [52–55] (figure 2). Furthermore, the dielectric constant (*ε*) reaches the normal value of 80 only at a distance of about 10 nm from the membrane while decreasing to about 6 in close proximity to it [56,57], notably owing to a reorientation of water molecule dipoles under the influence of the negative real charges by phosphates of membrane phospholipids. This liquid shielding sheath, which is schematically illustrated in figure 2, has a remarkable repulsive effect on ionized chemical species such as the polyanions ATP/ADP and NADH. This obstacle to membrane accessibility would explain why ion pumps such as sodium/potassium-transporting ATPase and sarcoplasmic/endoplasmic reticulum calcium ATPase have a molecular extensive arm with the binding site for ATP/ADP at almost 8 nm from the membrane (figure 2). Interestingly, the respiratory complex I presents a striking molecular extended arm with the binding site for the polyanion NADH at a distance from the membrane even greater than 10 nm. The existence of this considerable molecular arm is dictated by the need by complex I to create a redox interaction site with NADH, which has little chance of approaching the membrane. The polyanionic molecules such as Coenzyme A do not pass membranes, so much so that Acyl-CoA derivatives need the conversion to acyl-carnitine, and the enzyme that interacts with the Acyl-CoA is attached to the internal mitochondrial membrane and interacts with the substrates at a distance of about 5 nm from the membrane surface [58]. Notably, ATP is a polyanion itself.

It emerges that the proteolipid membrane is particularly isolated from the aqueous phase that lapses both sides and the reactions of the OxPhos (of considerable complexity) that occur in the membrane have no contact points with the aqueous phase (figure 2) excluding the beginning (capture of reducing equivalents from NADH from respiratory complex I) and the end (ATP synthesis by the ATP synthase). Consistently, it also emerges that there are two side arms operating at the beginning and the end of the process, respectively, the arm of the respiratory complex I and the F_1_ ATP synthase moiety.

## 5. A model for the aerobic ATP production in myelin sheath

The aerobic ATP production by myelin sheath was documented [59–62] and has been attributed to the expression of the ETC in its major dense line. Evidence showed that the aerobically produced ATP would be delivered to the axon through gap junctions of which the myelin is particularly rich [63], thus supporting the nerve conduction energetically [64]. Moreover, the F_1_ subunits would be present in significant amounts in myelin, as inferred from the titration with oligomycin which induces a quenching of the dye fluorescence RH-123 [59], and the considerable presence of lipid rafts containing mitochondrial components in myelin is noteworthy [5,65]. This suggests the existence of a route delivering the mitochondrial proteins to the sheath.

Interestingly, several studies performed with the freeze-fracture technique on the myelin highlighted numerous intramembranous particles on both sides of the major dense line [66–69], which decrease in rats with chronic streptozotocin-induced diabetes [67].

**Figure 3.**
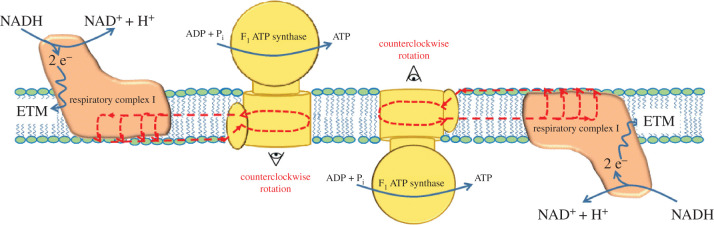
Schematic of a bi-faced ATP synthase arrangement in myelin, sustaining proton currents delivered by respiratory complex I, establishing a proton circuit entirely built inside a major dense line of myelin.

Moreover, in 1986, Gabriel *et al.* [66] observed that the myelin sheath surface of the peripheral nerve is covered by several particles, whose dimensions, 8.6 nm, coincide exactly with those of the dehydrated ‘spheres’ F_1_, suggesting that ATP synthase could be arranged with bi-faced orientation in the major dense line of the myelin sheath. Furthermore, the bi-faced orientation would conflict with the widely accepted hypothesis that it is the membrane potential that drives the synthesis of ATP in the major dense line of the myelin sheath. Particularly significant is the fact that the development of these intramembranous particles after the birth of the rat optic nerve [70] appears analogous to that described for ATP synthase in myelin [61]. However, this view implies an apparent biophysical discrepancy because the widely accepted model, which proposes that the membrane potential provides the driving force for ATP synthesis in mitochondria, does not apply to this bi-face arrangement. In fact, following Mitchell's theory, if a potential is active for the endergonic synthesis of ATP on one membrane side, the ATP synthase on the other side should perform the inverse reaction, hydrolysing ATP and establishing a futile cycle. To solve this problem, in figure 3, we propose a model in which the coupling between respiratory complex and ATP synthase could happen on a monolayer of the membrane complex (in the scheme is shown only the respiratory complex I for simplicity), as already reported in [36]. In particular, the scheme shows the single ATP synthase coupled through the H^+^ circuit with respiratory complex I oriented on its side. With this arrangement, they are active with both ATP synthase, regardless of their orientation. It should be noted that this scheme would allow for the central section of the circuit to be common to both the ATP synthase. This hypothesis is based on the fact that the circuit, built entirely inside a monolayer of the membrane, displays an H^+^ current that regards the charge but not the mass (Grotthuss mechanism) [71]. On the other hand, the experimental reconstruction of ATP synthase in liposomes has already provided a series of evidence in favour of the bi-faced orientation of this nanomachine [72].

Moreover, myelin appears a good ‘proton-sponge’ because it is rich in myelin basic protein, which, owing to its strong basic properties, could be particularly efficient in storing and disposing of protons.

## 6. Conclusion and perspectives

The universally accepted statement that mitochondria, intended as individually separated organelle, are the cell powerhouse bearing the exclusive ability to aerobically produce ATP for the cell was here reconsidered. Mitochondria and ER would have evolved early interactions rendering the mitochondrial aerobic ATP production more efficient along with the evolution. The endoplasmic and mitochondrial reticulum may be in close contact, forming a unit with a common functioning. Given this hypothesis, in some cell types, there is also the actual transfer of the OxPhos machinery from the IMM to the extra-mitochondrial sites, with the intermediation of the ER. An impressive amount of data shows that the extra-mitochondrial aerobic synthesis of ATP operates with great efficiency in the nervous system.

The active synthesis of ATP by the ER is an element of evolutionary connection, finding more and more credit for the theory that sees the ancestral bacteria, carriers of the OxPhos machinery, benefited by the development of the internal membranes present in the archaea that have then evolved into the eukaryotic cell to this symbiosis according to the mito-intermediate hypothesis [22] and, on the other hand, appear convincing that the ancestral eukaryotic cell has transmitted to the mitochondria the molecular devices to increase its own internal surfaces and, therefore, the efficiency of OxPhos. On the other hand, the mitochondria appear as the privileged site for the assembly of the complicated OxPhos machinery.

Although the present hypotheses attempt to answer several basic topics, the interaction between mitochondria and ER is well documented [43,44], the mechanisms involved in the OxPhos machinery transfer from mitochondria to the other cellular membranous structures remain to be clarified. Consistently, the ER lipid rafts contain components of OxPhos [8,19] and the plasma membrane expresses typical mitochondrial components [9]. In perspective, this is a question that needs to be answered adequately in future experimentation.

## Data accessibility

This article has no additional data.
